# Predominant Virulent IbA10G2 Subtype of *Cryptosporidium hominis* in Human Isolates in Barcelona: A Five-Year Study

**DOI:** 10.1371/journal.pone.0121753

**Published:** 2015-03-27

**Authors:** Remedios Segura, Núria Prim, Michel Montemayor, María Eugenia Valls, Carme Muñoz

**Affiliations:** 1 Servei de Microbiologia, Hospital de La Santa Creu i Sant Pau, 08041, Barcelona, Spain; 2 Servei de Microbiologia, Hospital Clínic de Barcelona, 08036, Barcelona, Spain; 3 Departament de Genètica i Microbiologia, Universitat Autònoma de Barcelona, Barcelona, Spain; University Hospital San Giovanni Battista di Torino, ITALY

## Abstract

**Background:**

*Cryptosporidium* infection is a worldwide cause of diarrheal disease. To gain insight into the epidemiology of the infection in a certain geographic area, molecular methods are needed to determine the species/genotypes and subtypes.

**Methodology/Principal Findings:**

From 2004 to 2009, 161 cryptosporidiosis cases were detected in two hospitals in Barcelona. Diagnosis was performed by microscopic observation of oocysts in stool specimens following modified Ziehl-Neelsen staining. Most cases (82%) occurred in children. The number of cases increased in summer and autumn. Molecular characterization of *Cryptosporidium* was performed in 69 specimens, and *C. hominis* and *C. parvum* were identified in 88.4% and 10.1% of the cases, respectively. *C. meleagridis* was detected in one specimen. Subtyping based on the *gp60* polymorphism showed six subtypes, four *C. hominis* and two *C. parvum*. Subtype IbA10G2 was the most prevalent subtype corresponding to 90% of all *C. hominis* isolates. This is the first report on the distribution of specific *Cryptosporidium* subtypes from humans in Spain.

**Conclusions/Significance:**

In our geographic area, the anthroponotic behavior of *C. hominis*, the lower infective dose, and the higher virulence of certain subtypes may contribute to the high incidence of human cryptosporidiosis caused by the IbA10G2 subtype. Further studies should include populations with asymptomatic shedding of the parasite.

## Introduction

*Cryptosporidium* infection is a major cause of diarrheal disease worldwide. Differences in the clinical manifestations depend on characteristics of both the human host and the parasite [1–3]. Cryptosporidiosis affects all age groups but has a major impact in children and immunosuppressed populations. Besides diarrhea, symptoms may include vomiting, abdominal pain, or other gastrointestinal complaints/problems. Diarrhea is usually self-limited but it may become chronic under conditions of strong immunosuppression [1]. As *C. hominis* and *C. parvum* have different infective doses and virulence, their potential to cause outbreaks also differs [1–3]. Asymptomatic carriage of *Cryptosporidium* oocysts has also been described [1,4,5].

Human cryptosporidiosis infection is acquired though the intake of *Cryptosporidium* oocysts. The main transmission routes are water, food, person-to-person and animal-to-person and the disease may present in outbreaks or as sporadic cases [1]. The multiple *Cryptosporidium* reservoirs and the existence of several species that can cause the disease in humans irrespectively of their immunological status also contribute to the epidemiological complexity of this genus [1].

The species most commonly involved in human cryptosporidiosis are *C. hominis* and *C. parvum*. The geographic distribution of both these species varies even within the same country [3,5,6–10]. However, the environmental source of infection, whether a rural environment or an urban area, seems to be the main factor contributing to this variation [3,8,10,11]. *C. hominis* has classically been considered to be mainly anthroponotic while *C*.*parvum* is considered to have zoonotic transmission [3]. *C. meleagridis*, *C. felis*, *C. canis*, *C. muris*, *C. suis* and *C. andersoni* are also zoonotic species only occasionally involved in human cases [3,12].

*Cryptosporidium* species/genotypes can not be differentiated using traditional diagnostic methods, and therefore molecular methods must be used [3]. Within each species and based on the variations in the polymorphism region of the 60-kDa glycoprotein (gp60) gene, subtypes have been described and classified into few subtype families [2,3]. Knowledge on the circulation of particular subtypes may help understand the complex biology of this parasite. The aim of this study was to gain insight into the distribution of subtypes related to human cryptosporidiosis in our area. To our knowledge, this is the first report on the distribution of specific *Cryptosporidium* subtypes from humans in Spain.

## Methods

### Ethics Statement

The Ethics Committee of Hospital de la Santa Creu i Sant Pau approved the research (approval number: IIBSP-CRY-2014-26) and waived the need for consent. The samples were anonymized.

### Data and specimen collection

A total of 161 cryptosporidiosis cases were reported in two hospitals in Barcelona, Hospital de la Santa Creu i Sant Pau (HSCSP) and Hospital Clínic (HCP), from 2004 to 2009. Diagnosis was performed by microscopic observation of oocysts in diarrheal stool specimens following modified Ziehl-Neelsen staining [13]. Stool specimens were kept at -80°C until molecular characterization was performed. Only one sample was collected per patient. Epidemiological data (sex, age, nationality, date of collection and immune status) were collected retrospectively from patients diagnosed in HSCSP. As information from HCP was partially biased due to lack of knowledge about the origin of the samples, these data were not considered for epidemiological purposes. The seasonal distribution of cases was compared to data from the national weekly epidemiology report (http://www.isciii.es/jsps/centros/epidemiologia/boletinesSemanal.jsp) provided by the Instituto de Salud Carlos III (ISCIII, Madrid, Spain). Sixty-nine stool samples were available from the 161 cases for further molecular characterization, 55 from HSCSP and 14 from HCP. Given the low number of cases, statistical analysis was not performed.

### Molecular analysis

Nucleic acid was extracted from stool samples using the QIAamp Stool Mini Kit (Qiagen GmbH, Hilden, Germany), according to the manufacturer’s instructions. The molecular characterization of the different species/genotypes of *Cryptosporidium* was performed using PCR–restriction fragment length polymorphism (RFLP) analysis of the small subunit rRNA genes, as previously described [14].

Subtyping was performed by sequence analysis of the *gp60* gene [15]. Sequencing reactions were performed in Macrogen Inc. (Seoul, Korea). Nucleotide sequences were analyzed with Seqman II (DNASTAR, Madison, USA) and compared to those in the GenBank using BLAST (http://blast.ncbi.nlm.nih.gov/Blast.cgi).

## Results

**Fig 1:**
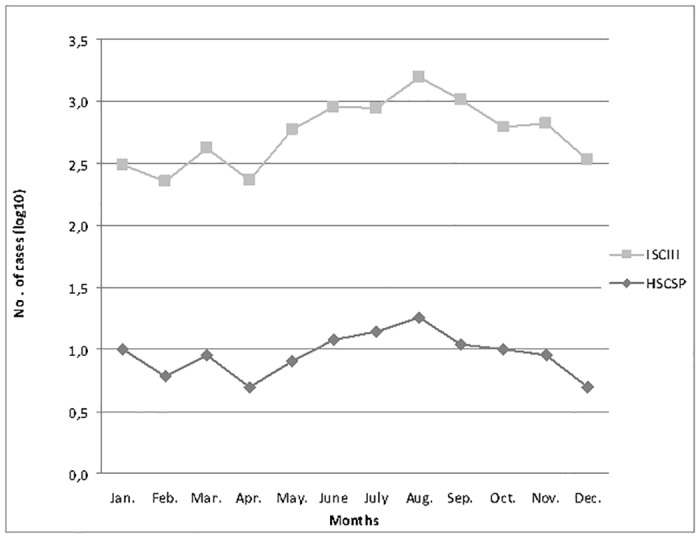
Monthly distribution of cryptosporidiosis cases at HSCSP and in Spain according to ISCIII (2004–2009).

A total of 161 sporadic cryptosporidiosis cases were reported from the two hospitals (HCP and HSCSP) between 2004 and 2009. Only the 117 cases from HSCSP were used for epidemiological studies. Of these, 75 cases (64%) corresponded to males. Regarding the age of patients, 96 cases (82%) were diagnosed in children younger than 16 years of age. Fig. 1 shows the monthly distribution of the cryptosporidiosis cases identified in HSCSP and the cryptosporidiosis incidence reported by the ISCIII. Cryptosporidiosis cases increased during the summer and autumn months in both institutions, with the highest number accumulating in August: 27.7% (18/65) in HSCSP and 23% (88/382) in the ISCIII. From June to October, the incidence was 55.6% (65/117) in HSCSP and 50.3% (382/759) in ISCIII.

**Fig 2:**
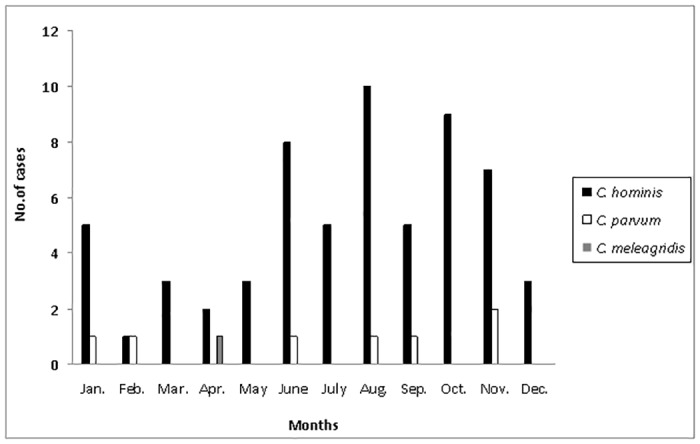
Monthly distribution of the cryptosporidiosis cases molecularly characterized (n = 69) and the species/genotype involved (2004–2009).

Only 69 stool samples were available for further molecular characterization, 72% of which corresponded to children. All samples were collected from autochthonous patients, five of whom had recently traveled outside Europe (India, South America and Africa). Eleven samples belonged to immunosuppressed patients, seven HIV-infected adults, one patient with heart transplantation and three patients with hematological disease, two of whom were children (Table 1). Nine out of 69 patients were co-infected with another enteric pathogen, i.e. *Campylobacter jejuni* (n = 3), *Salmonella enterica* (n = 2), *Giardia lamblia* (n = 2), *Chilomastix meslini* (n = 1) and *Dientamoeba fragilis* (n = 1). Molecular identification of the isolates at species/genotype level showed the presence of *C. hominis* and *C. parvum* in 61 (88.4%) and seven cases (10.1%), respectively (Table 1). *C. meleagridis* was detected in a specimen from an HIV-infected adult patient. The monthly distribution of the characterized strains followed the same pattern as the global distribution for all cases collected at both institutions (Fig. 2).

**Table 1:** Characteristics of the cases of cryptosporidiosis molecularly characterized (n = 69).

Characteristics		Species/genotype (n)		
		*C*. *hominis* (61)	*C*. *parvum* (7)	*C*. *meleagridis* (1)
Age				
	Children (0–5 years)	32	2	-
	Children (>5 years)	14	2	-
	Adults (≥18 years)	15	3	1
Immunosupression^a^				
	HIV	6	-	1
	Hematological disease	3	-	-
	Heart transplantation	-	1	-

^a^All immunosupressed patients were adults except two children with hematological disease.

Subtyping was successfully performed by *gp60* gene sequencing on 67 of the 69 isolates, showing 100% homology with sequences from the GenBank (Table 2). The remaining two isolates could not be subtyped, one *C. hominis* strain and the only *C. meleagridis* strain isolated. Phylogenetic analysis showed six subtype families, four *C. hominis* (Ib, Id, Ie, If) and two *C. parvum* (IIa,IId). A total of nine subtypes were detected. Subtype IbA10G2 was the most prevalent, corresponding to 90% of all *C. hominis* isolates (54/61). All subtype IbA10G2 isolates were obtained from patients who had not traveled. Regarding age distribution, the IbA10G2 subtype was detected in 86% of specimens in children (43/50) and 58% of specimens in adults (11/19). Non-Ib subtypes of *C. hominis* (6/61) corresponded to IdA15G1, IeA11G3T3, IfA12G1 and IfA14G1, and were sparsely detected in this study. Three of these non-Ib subtype isolates were linked to foreign travels. Six out of seven isolates of *C. parvum* isolates belonged to subtype family IIa, and the IIaA15G2R1 subtype was detected in four of these.

**Table 2:** Species/genotypes and subtypes of *Cryptosporidium* characterized in this study (n = 69).

*Cryptosporidium* species/genotype (n)	Subtype	No. of positive specimens (n)
***C*. *hominis*** (61)	Ib A10G2	54
	Ie A11G3T3	2
	If A12G1	2
	Id A15G1	1
	If A14G1	1
	ND	1
***C*. *parvum*** (7)	IIa A15G2R1	4
	IIa A18G2R1	1
	IIa A20G1R1	1
	IId A21G1	1
***C*. *meleagridis*** (1)	ND	1

ND: No data

## Discussion

*C. hominis* was the species/genotype most frequently detected in the present study. This supports an inter-human transmission as the main route, in agreement with other studies performed in urban areas [3,9]. Our results showed a low prevalence of *C. parvum* and other zoonotic species in our geographic area, unlike a recent report from rural and peri-urban areas from other regions in Spain [10].

In our study, most cases of cryptosporidiosis (82%) were detected in children, in accordance with other studies [1]. The susceptibility of the human host is an important factor for it contributes to the severity of clinical manifestations. Severity is higher in children and immunocompromised patients [1].

Our results showed an increase of cryptosporidiosis cases during the summer-autumn period although the low number cases per month were not enough to determine a seasonal distribution. However, these results were in agreement with data from national weekly epidemiology report (ISCIII) and reports from Public Health Services from other European countries [9,16]. The higher incidence in summer may be explained by a waterborne transmission related to the use of recreational water, in agreement with several waterborne outbreaks previously reported in warm seasons [3,17]. The cases reported in autumn may be due to contaminated drinking waters or to a person-to-person transmission, especially linked to children returning to school [18–20]. Another important issue contributing to the epidemiological complexity of this parasite is that *Cryptosporidium* infections may remain asymptomatic. The epidemiology of carriers is not well-established but asymptomatic shedding may have a key role as a source of infection [4,5].

In the present study, the genetic diversity among human isolates of *C. hominis* had low heterogeneity as most of them belonged to the Ib family. All isolates belonging to this family corresponded to the subtype IbA10G2 and were from autochthonous patients. As previously described, subtype IbA10G2 has a global distribution and it has been related to both outbreaks and sporadic infections in industrialized nations [3,21]. In contrast, a higher diversity has been reported in non-industrialized countries [21]. This is in correlation to our results in which all patients with a travel history outside Europe had non-Ib subtypes. A further issue when considering the geographic distribution is the different virulence reported within *C. hominis* subtypes [3]. Despite all subtypes causing diarrhea, the subtype family Ib has been shown to be the most virulent, a fact that might also contribute to the higher number of isolates [3].

The real epidemiology of human cryptosporidiosis is biased due to several factors. Until 2009 cryptosporidiosis was not a notifiable infection in Spain and therefore not all cases were reported to the weekly epidemiology report (ISCIII) [22]. Contributing to the fore mentioned bias, only severe and persistent cases of diarrhea are consulted. In those cases, microbiological studies are usually performed but *Cryptosporidium* should be required specifically under clinical suspicion [6]. Additionally, the real prevalence in humans is unknown because the carriers are not usually considered. The bias of the real epidemiology in humans is even more relevant when it comes to subtypes. Knowledge of the subtype distribution in carriers would help understand whether higher detection of a particular subtype is due to its higher virulence or because it is the most prevalent in an area. As previously mentioned, the IbA10G2 subtype was the most frequently detected but carriers were not studied in the present report. This subtype has been recently shown to be the most virulent subtype [2,3]. The higher fitness of the IbA10G2 subtype is considered to be related to its spread and higher detection, and it may contribute to the selection of virulent strains [2].

This study brings new insights in the subtypes contributing to human cryptosporidiosis in our geographic area. A recent Spanish report describes the use of high-throughput techniques for *Cryptosporidium* characterization on the basis of subtype families [23]. However, highly discriminatory methods for characterization of different subtypes within each family, including rare variants, are required for a better understanding of the transmission dynamics of this parasite [2,3,24], as in the present work. The combination of the *C. hominis* behavior, which is mainly anthroponotic, the lower infective dose and the higher virulence of certain subtypes may contribute to the high incidence of human cryptosporidiosis caused by IbA10G2 subtype in this area. To have more accurate information on the real prevalence of subtypes in our area, further studies should also include population with asymptomatic shedding of the parasite.
